# A values-driven approach to vaccine hesitancy conversations

**DOI:** 10.4102/safp.v64i1.5419

**Published:** 2022-01-31

**Authors:** Zilla M. North, Arnold T. Smit, Louis S. Jenkins

**Affiliations:** 1 George Sub-District, Garden Route District, Western Cape Department of Health, George, South Africa; 2 University of Stellenbosch Business School, Stellenbosch University, Cape Town, South Africa; 3 Department of Family and Emergency Medicine, Faculty of Medicine and Health Sciences, Stellenbosch University, Cape Town, South Africa; 4 Department of Primary Health Care, Faculty of Health Sciences, University of Cape Town, Cape Town, South Africa; 5 Department of Family and Emergency Medicine, Western Cape Department of Health, George Hospital, George, South Africa

**Keywords:** COVID-19, vaccine, hesitancy, values, ethics, conversations

## Abstract

South Africa recently experienced the third wave of the coronavirus disease 2019 (COVID-19)pandemic. Social media is flooded with polarised conversations, with opinions for and against severe acute respiratory syndrome coronavirus 2 (SARS-CoV-2) vaccines. Many people are hesitant, and some are strongly opposed to vaccination. Vaccine hesitancy must be understood in historical, political and socio-cultural contexts. The aim of this study was to offer a values-driven approach to vaccine hesitancy conversations. It focusses on ethical dilemmas forthcoming from values violations, interrogating the personal and institutional scripts and rationalisations that prevent resolution, and offering ways of re-scripting these. Values-driven conversations provide safe spaces for vaccine-hesitant individuals to voice their reservations. The manner in which conversations are conducted is as important as the contents being discussed. Healthcare professionals are trusted by the public and should use ways of conversing that do not erode this trust. Creating respectful, compassionate platforms of engagement and incentivising vaccination are important measures for change in vaccine perspectives.

## Introduction

South Africa (SA) has recently emerged from the third wave of the coronavirus disease 2019 (COVID-19) pandemic. The impact has been devastating, with over 4 million deaths reported worldwide and 89 562 recorded deaths nationally (Nov 2021).^1^ The negative impact on the economy and the additional burden on the healthcare sector are ongoing. Social media is flooded with heated conversations that has caused polarisation of people and groups, with a spectrum of opinions for and against various treatment modalities and the severe acute respiratory syndrome coronavirus 2 (SARS-CoV-2) vaccines.

Vaccination roll-out in SA started in May 2021. To date (19 Nov 2021), 40% of the adult population have been fully vaccinated.^1^ One in four participants in a national survey remained hesitant about getting vaccinated and one in 15 were strongly opposed to vaccinations, with a large gap between the proportion of people indicating their willingness to be vaccinated and those who had done so.^2^ The contributing factors to vaccine hesitancy relate to uncertainty and distrust.^3^ Vaccine acceptance in low- and middle-income countries (LMICs) was mostly explained by an interest in personal protection against COVID-19, while concerns about side effects was the most common reason for hesitancy, with health workers being the most trusted sources of guidance about COVID-19 vaccines.^4^

Complex, context-specific factors that vary across time, place and different vaccines, influenced by issues of complacency, convenience, confidence and sociodemographic contexts, contribute to vaccine hesitancy.^5,6^ Misinformation, conspiracy theories and structural factors, such as health inequalities, socioeconomic disadvantages, systemic racism and barriers to access, are further key drivers of poor vaccination uptake.^7^

Vaccine hesitancy is an individual behaviour influenced by knowledge or past experiences.^8^ It must be understood in the historical, political and socio-cultural context in which vaccination occurs. Trust in the system that delivers vaccines, in health professionals who recommend and administer the vaccines, in policy decision makers and in media information mediate the impact of these factors on vaccine hesitancy.^8^ The aim of this study was to offer a practice-oriented approach to vaccine hesitancy conversations through a values-driven model.

## A values-driven conversation approach

We propose an approach that was originally pioneered by Dr Mary C. Gentile, called ‘Giving Voice to Values’ (GVV).^9^ This is an innovative approach to values-driven leadership (VDL) development in business education and the workplace. Giving Voice to Values starts from the premise that most people want to act on their values, but they also want to feel that they have a reasonable chance of doing so effectively and successfully. This approach is about raising those odds. Rather than a focus on ethical analysis, the GVV curriculum focuses on ethical implementation and asks the question: ‘[W]hat if I were going to act on my values? What would I say and do? How could I be most effective?’.

Informed by Gentile’s GVV approach, alongside notions of relationality from African and 20th century European philosophical traditions, a VDL programme was developed, offering a unique alternative to rationalistic Western approaches to moral education and leadership development.^10,11^ The original programme was initiated by the Academy of Business in Society (ABIS), a global network of over 100 companies and academic institutions whose expertise, commitment and resources are leveraged to invest in a more sustainable future.^12^ The ABIS collaborated with its business school members in SA, Kenya and Egypt to establish a three-pronged leadership development programme focusing on responsible leadership, sustainable development and ethical business conduct. Eventually coined as VDL, the programme helps people understand how universal values of honesty, respect, responsibility, fairness and compassion apply to relationships at home, at work and in society.^11^ Values-driven Leadership focusses on ethical dilemmas emanating from values violations in the professional workplace, interrogating the personal and institutional scripts and rationalisations that prevent such dilemmas from being resolved, and offering ways of re-scripting these.

A version of the VDL for healthcare professionals has been developed by the authors and presented in the Garden Route district. Since February 2019, more than 100 healthcare professionals from different disciplines completed the programme. Values-driven conversations should provide safe spaces in which vaccine hesitant individuals are afforded the opportunity to voice both their factual (even if misinformed) and moral (even if inappropriate) reservations. The manner in which such conversations are conducted is as important as the contents being discussed. Inasmuch as values are important guides for ethical action, they are also the premises on which respectful conversations and hence relationships are based. Such conversations are grounded in four principles (see Table 1).^11^

**TABLE 1:** Principles of a values-driven approach to difficult conversations.

Number	Principle	Explanation	Guidelines to facilitate conversations
1.	Active listening	The context in which a sensitive conversation happens, is as important as the contents of the conversation itself. Active listening is not premised on persuasion but understanding.	Listening while maintaining the dignity of the other, the speaker Suspending judgement of what the other is saying Support to the other to speak their whole truth/experience Guarantee confidentiality for the conversation to happen in a safe space Learning while listening, which includes exploring the concepts discussed through active questioning and engagement
2.	Realising that emotions are signifiers of values confrontations	We live relationally embedded lives and the extent to which our values are honoured or violated determines much of our emotional wellness. Universal values can be summarised as honesty, fairness, responsibility, respect and compassion. Situations in which these values are experienced to be violated, generate different negative emotions.	What is the situation that you are in? Who else is involved? What makes you happy, angry, frustrated, or sad about the situation? What values do you experience as being important or potentially violated by how you are treated? How do you feel you are being treated for having a different view on the matter?
3.	Understanding the excuses or rationalisations that bind us to inaction. These are often expressed as ‘scripts’.	Resolving a values violation or ethical dilemma is not natural for most people. We tend to offer rationalisations or excuses for not doing the right thing.	Standard practice: I do not need to be an exception to the rule. There are many others who seem to feel or do the same. Materiality: The consequences of me not complying are negligible, it will not harm anyone else. Locus of responsibility: Do not talk to me about responsibility. Everyone is free to decide for themselves. Locus of loyalty: If I act to please others, I feel I am sacrificing my own convictions.
4.	Building our moral muscle to rescript and motivate for change (using the tools available, including our position, relationships, network, governance processes and asking the right questions).	We need to move away from excuses or rationalisations to a position where we can get the right thing done for the right reasons. Well-phrased and inviting questions may help to make meaningful conversations possible.	From standard practice to best practice: What kind of information will help you to make a fully informed and responsible decision? From minimising materiality to transparency: To whom does your behaviour matter? What do they need to know about you? From deferring responsibility to action: What action can you take responsibility for? What form may this take? From subjective self-interest to objectivity: If you maintain your position, how can you best enable others to continue to have a meaningful relationship with you?

## Values-driven conversations on vaccine hesitancy

Using this approach, when engaging vaccine hesitancy, the following become evident:

- When we listen to conversations with people who are vaccine hesitant, we learn the following^6,7^:
  ■ Vaccine hesitancy exists on a continuum from active demand for vaccines to complete refusal of all vaccines. Desist from putting people in binary boxes.
  ■ Hesitancy is not directly related to vaccine uptake, which causes difficulty in measuring it.
  ■ Hesitancy to newer vaccines is more rampant than to older vaccines.
  ■ ‘Local vaccine culture’ is influenced by local health service experiences, information available and religion.
  ■ Social media perpetuates the experienced relevance of vaccine scares and controversies that may already have been resolved.
  ■ Risk perception is complicated and not influenced by scientific information on risk, as this is often too complex to understand and interpret practically.
  ■ ‘They are hiding something’ is an overwhelming suspicion.
- Vaccine hesitant individuals express their values-based scripts as follows:
  ■ Honesty: The truth is hidden; there is not enough information available. Local practical data is not available.
  ■ Respect: I am not your guinea pig. I’m not gullible, you’re not going to get rich off me.
  ■ Fairness: It’s not fair to vaccinate me to ensure someone else stays well if we are not sure of the long-term effects on my health.
  ■ Responsibility: Everyone should take responsibility for their own health. God is responsible for deciding who lives or dies. I am responsible for ensuring my family is safe and I don’t have enough information to satisfy my risk perception.
  ■ Compassion: I love my family. I will never be able to forgive myself if something happened to me/them because of vaccines.
- Consequently, vaccine-hesitant individuals rationalise their position as follows:
  ■ Standard practice: Many others, even experts, do not trust these vaccines. Why should I be different?
  ■ Materiality: What does it matter if I’m not vaccinated? Herd immunity will still happen without me risking my life. I won’t get that sick from COVID anyway. We live in an isolated area; it doesn’t matter if we are vaccinated or not.
  ■ Locus of responsibility: It’s your responsibility to prove to me that the vaccine is safe. My responsibility to my community by taking the vaccine is misstated/misrepresented. You are responsible to provide me with healthcare despite what I choose to do.
  ■ Locus of loyalty: my loyalty is to my own health and my family, not the government or big pharma.
- In response to what we learn from the above, a number of re-scripting possibilities exist (see Box 1). It is important to remember that hesitancy melts away slowly. It is not an on-off switch. Patience and continued engagement are important. Individuals may not remain hesitant forever if they are so presently. One cannot hope for a silver bullet or perfect message that will finally get through to everyone. The question is, therefore, how can we conduct a conversation that may help a person to explore the benefits of vaccination and come to an informed decision?

BOX 1 Re-scripting possibilities.

- From standard practice to best practice: If someone’s vaccine hesitancy is based on the example set by others and previous experience, it will be helpful to ask about sources of information that will clarify the inherent risks to help them make a well-informed decision. What information about both sides of the vaccine conversation have you obtained thus far? Who are the voices you trust most? What additional information can I help you with? What will give you confidence and courage to get vaccinated?
- From minimising materiality to transparency: If someone’s vaccine hesitancy seems to be borne from the minimisation of risk, it may be helpful to ask questions that will help them see the bigger picture about themselves in relation to others. How important is it for you that we all gain some form of immunity, whether natural or vaccine induced, to COVID-19? What difference does it make to how you pursue immunity, to yourself, and to others? What are the risks related to pursuing natural induced immunity to self and others?
- From deferring of responsibility to action: If someone’s vaccine hesitancy is based on making it other people’s problem, then questions that may support reflection on personal responsibility may be helpful. What do you normally do when you learn about a potentially life-threatening and contagious disease in the community? Have you taken preventative measures in such situations before? In these situations, have you been concerned about your family, friends and colleagues’ safety as well and encouraged them to take precautions too? What role did medicines and vaccinations play in this regard? How can you best provide evidence of being a responsible person who acts in the interest of yourself and the people that you care about most?
- Form subjective self-interest to objectivity: A person might feel torn between opposing loyalties. If someone seems to be caught in the middle of loyalty to their own convictions, even if skewed or misinformed, and the pressure of others, about getting vaccinated, questions around their most precious relationships might be helpful. Who are the people that matter most to you? How would you like to relate to them? What will be most helpful for you and them to have the most connected, natural and safest interaction during this difficult time of the pandemic? What role do you think can you being vaccinated play in normalising these precious relationships?

## Discussion

It is clear that in SA with its diverse cultures, languages, levels of education and religious inclinations, respectful conversations and building trust are important when addressing complex issues. Healthcare professionals are trusted sources of vaccine information and can influence vaccination rates by working alongside local authorities, faith leaders and communities by facilitating engagement, guiding household decision makers and making vaccine recommendations.^13^ Actively listening to each other, without judgement or interruption, considering these nuanced concerns, is a skill that all health professionals would do well to master. Healthcare professionals fulfil an important role by being not only a part of the scientific community but also being trusted members of the public. It is important to use ways of conversing that do not erode the trust earned through professional and ethical practice.

Creating respectful, compassionate platforms of engagement and incentivising vaccination by highlighting the benefits will be important measures for bringing about change in perspective. When a significant portion of the population is hesitant to a preventative measure that is strongly recommended and proven to work, it is important to understand what underlying values violations are being experienced and to address these earnestly and honestly. The disruptive changes in the last few months, many enforced by mandates, have resulted in excuses for inaction, most likely in an attempt to regain a feeling of control and self-determination. It is understandable that many vaccine hesitant excuses will be encountered. Respectfully supporting people through change will build trusting relationships that will position us better for inevitable future changes and disruptions. This will help to explain the excuses that bind people to inaction.

We know from our own historical experience that truth and reconciliation can bridge wide divides in viewpoints and where this succeeds, we reap the rewards of shared empathy and solidarity. As we practise how to assist patients to rescript and motivate for change, we hone an important tool for addressing inaction on multiple levels. It may not be necessary to mandate vaccines. We may achieve surprisingly more by ensuring that our communication is respectful, honest, responsible, fair and compassionate. A SA report supports this viewpoint by recommending clear leadership, transparency and thoughtful communication around responses to the COVID-19 pandemic and vaccines in order to enhance public trust and vaccination acceptance.^14^

## Conclusion

The aim of this study was to describe a values-driven model that could facilitate conversations around vaccine hesitancy. The vaccination debate pulls science and values together in the same space. Instead of overpowering the hesitant with either science or morals, a values-driven conversation approach may result in individuals taking a well-informed and confident decision in which personal dignity and the common good of all may both be preserved.

## References

[1] National Department of Health. COVID-19 public dashboard [homepage on the Internet]. [cited 2021 Nov 20]. Available from: https://sacoronavirus.co.za/latest-vaccine-statistics

[2] Burger R, Maughan-Brown B, Köhler T, et al. A shot in the arm for South Africa – Increased openness to accepting a COVID-19 vaccine: Evidence from NIDSCRAM waves 4 and 5 [homepage on the Internet]. 2021 [cited 2021 Oct 06]. Available from: https://cramsurvey.org/wp-content/uploads/2021/07/2.-Burger-R.-Maughan-Brown-M.-Kohler-T.-English-R.-_-Tameris-M.-2021-Increased-openness-to-accepting-a-COVID-19-vaccine-is-a-shot-in-the-arm-for-South-Africa-Evidence-from-the-NIDS-CRAM-Wave-5-Survey.pdf

[3] Loomba S, De Figueiredo A, Piatek SJ, et al. Measuring the impact of COVID-19 vaccine misinformation on vaccination intent in the UK and USA. Nat Hum Behav. 2021;5:337–348. 10.1038/s41562-021-01056-1 33547453

[4] Solís Arce JS, Warren SS, Meriggi NF, et al. COVID-19 vaccine acceptance and hesitancy in low- and middle-income countries. Nat Med. 2021;27:1385–1394. 10.1038/s41591-021-01454-y 34272499 PMC8363502

[5] Plotkin S. History of vaccination. Proc Natl Acad Sci USA. 2014;111(34):12283–12287. 10.1073/pnas.1400472111 25136134 PMC4151719

[6] Dubé E, Laberge C, Guay M, et al. Vaccine hesitancy. Human Vaccines Immunotherapeut. 2013;9(8):1763–1773. 10.4161/hv.24657 PMC3906279 23584253

[7] Razai MS, Chaudhry UAR, Doerholt K, et al. Covid-19 vaccination hesitancy. BMJ. 2021;373:n1138. 10.1136/bmj.n1138 34016653

[8] Larson HJ, Cooper LZ, Eskola J, et al. Addressing the vaccine confidence gap. Lancet. 2011;378(9790):526–535. 10.1016/S0140-6736(11)60678-8 21664679

[9] Gentile MC. Giving voice to values: How to speak your mind when you know what’s right. New Haven, CT: Yale University Press; 2010.

[10] Gentile MC. Keeping your colleagues honest. Harv Bus Rev. 2010;88(3):114–117.

[11] Pérezts M, Russion J, Painter M. This time from Africa: Developing a relational approach to values-driven leadership. J Bus Ethics. 2020;161(4):731–748.

[12] The Academy of Business in Society (ABIS) [homepage on the Internet]. [cited 2021 Nov 20]. Available from: https://www.abis-global.org

[13] Peterson P, McNabb P, Maddali SR, et al. Engaging communities to reach immigrant and minority populations: The Minnesota Immunization Networking Initiative (MINI), 2006–2017. Public Health Rep. 2019;134(3):241–248. 10.1177/0033354919834579 30912998 PMC6505329

[14] Cooper S. Report: Covid-19 vaccine hesitancy in South Africa: Summary of existing studies [homepage on the Internet]. Cochrane, South African Medical Research Council Centre for Social Change and Human Sciences Research Council’s (HSRC). 2021 [cited 2021 Oct 06]. Available from: https://sacoronavirus.co.za/wp-content/uploads/2021/04/Report_Covid-19-vaccine-hesitancy_SA-studies_1April2021.pdf (accessed 6 October 2021).

